# Spectroscopic Insights into Lysozyme Structural Modulation by Polyphenols: A Combined FTIR and VCD Study

**DOI:** 10.3390/molecules31162872

**Published:** 2026-08-17

**Authors:** Karolina Musiał, Katarzyna Cieślik-Boczula

**Affiliations:** Faculty of Chemistry, University of Wrocław, Joliot-Curie 14, 50-383 Wrocław, Poland

**Keywords:** VCD spectroscopy, synergistic effect, antagonistic effect, quercetin, resveratrol, caffeic acid

## Abstract

Protein fibril formation is of considerable interest due to the association of fibrils with many neurodegenerative diseases as well as non-neuropathic amyloidosis. In this study, the effects of individual phenolic compounds and their mixtures—including caffeic acid (CC), quercetin (Que), and resveratrol (Res)—on the fibrillation of lysozyme (Lys) were investigated using Fourier transform infrared (FTIR) and vibrational circular dichroism (VCD) spectroscopy. These phenolic compounds are known as potent modulators, particularly inhibitors, of fibrillation in various proteins and peptides. While their anti-fibrillar activity has been extensively studied via the Thioflavin T (ThT) fluorescence assay, here FTIR and VCD spectroscopy are employed for a comprehensive, label-free investigation of their effects. The results indicate that CC, Que, and Res influence Lys fibrillogenesis in ways that depend on the phenol concentration, their combination (mixing ratios), and the experimental conditions. In particular, Lys was observed to form left-handed fibrils under these conditions in the presence of these phenolic compounds, as evidenced by the VCD signal. Furthermore, a comparison was made of the inhibitory effects of the individual phenols and their mixtures on Lys fibril formation: CC and Res were more effective inhibitors than Que. Interestingly, an equimolar mixture of CC and Res exhibited a strong synergistic inhibitory effect on fibrillation, whereas the combination of CC with Que showed an antagonistic effect. Overall, this study provides deeper insights into how specific polyphenolic compounds influence protein fibrillation processes, offering valuable information for developing therapeutic interventions targeting protein aggregation-associated disorders.

## 1. Introduction

Misfolding and aggregation of proteins into amyloid fibrils are linked with a variety of diseases. Protein aggregates can impair cellular function and contribute to neuronal dysfunction or death, as supported by recent human-neuron and in vivo studies [1,2,3,4,5,6]. Recent work further shows that reducing aggregate burden or aggregate-associated toxicity can improve pathological or functional outcomes in experimental models [5,6,7,8,9,10]. These observations support strategies aimed at limiting amyloid self-assembly, reducing aggregate toxicity, or promoting aggregate clearance.

Rather than assigning amyloid assembly to a single interaction type, current evidence indicates that aromatic side chains can contribute together with hydrophobic, hydro-gen-bonding, and other non-covalent interactions to peptide self-assembly and fibril organization [11,12,13]. In parallel, natural phenolic compounds have been shown to modulate amyloid fibrillation in a strongly compound-, concentration-, and condition-dependent manner [14,15,16,17,18,19,20]. Accordingly, the anti-fibrillar effects of three red wine polyphenols, caffeic acid (CC), quercetin (Que), and resveratrol (Res), on lysozyme (Lys) fibrillogenesis were examined in detail. Computational docking studies have shown that all three of these phenols can bind to regions of hen egg-white Lys that are directly involved in forming the fibrillar core [14]. These observations support the evaluation of CC, Que, and Res as modulators of Lys fibrillation. Furthermore, a multi-drug treatment strategy is currently being considered in the search for effective therapies for cancer and other diseases. In this context, a synergistic interaction—where two or more biologically active compounds mutually enhance each other’s anti-fibrillar activity—could lead to increased efficacy of combination treatments.

**Figure 1:**
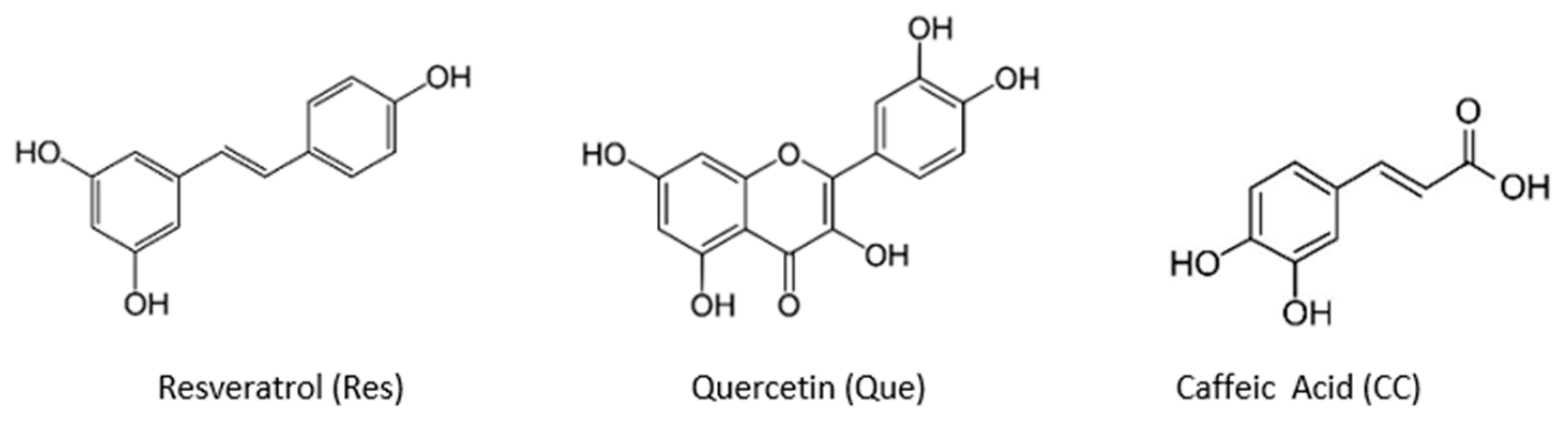
Chemical structures of caffeic acid (CC), quercetin (Que), and resveratrol (Res).

In this work, an investigation is conducted into the ability of three structurally different red wine polyphenols (CC, Que, and Res), whose chemical structures are presented in Figure 1, alone and in binary or ternary mixtures, to modulate the secondary structure of Lys and its self-assembly into fibrillar aggregates. Fourier transform infrared (FTIR) and vibrational circular dichroism (VCD) spectroscopy are employed to evaluate potential synergistic or antagonistic effects of the tested phenols, while transmission electron microscopy (TEM) is used to confirm the presence of fibrillar aggregates. FTIR spectroscopy (particularly the analysis of the amide I vibrational region) is a widely used method for defining protein secondary-structure content [17,21,22,23,24,25]. VCD spectroscopy is an excellent tool for studying the formation, growth, structural polymorphism, and chirality of protein fibrils [17,26,27,28,29,30,31,32,33]. Notably, unlike dye-based assays such as Thioflavin T (ThT) fluorescence, FTIR and VCD provide direct label-free insights into secondary-structure changes and supramolecular organization of protein aggregates. In addition, VCD can detect fibril chirality, distinguishing left- vs. right-handed supramolecular structures by the sign pattern of the VCD couplet. A positive VCD band around 1620 cm^−1^ paired with a negative band around 1610 cm^−1^ indicates left-handed twisted fibrils, whereas an inverted negative–positive couplet would signify right-handed fibrils [28]. Our results confirm the preservation of the left-handed chirality of Lys fibrils in the presence of all investigated phenolic compounds.

## 2. Results

### 2.1. Structural Polymorphism of Lysozyme in a DMSO-Rich Solution Under Thermal Treatment

Hen egg-white Lys is known to rearrange its native structure into fibrillar aggregates under acidic and/or thermal stress [30,34,35]. Using DMSO as a cosolvent can lower the thermal stability of Lys and promote partial unfolding, thereby facilitating structural reorganization under less severe thermal conditions [28,36]. On the other hand, it should be mentioned that these conditions differ substantially from physiological protein aggregation. Recent thermodynamic, calorimetric, and spectroscopic studies confirm that the composition of DMSO–water mixtures strongly affects preferential solvation, thermal stability, and unfolding of Lys [36,37,38,39,40,41]. DMSO, a molecule with both polar and apolar characters, can perturb the solvent environment and alter the balance of interactions stabilizing the protein [36,37,38,39,40]. Torreggiani et al. [41] showed that a high concentration of DMSO in water at room temperature acts as a denaturant, causing about a 10% loss of Lys enzymatic activity accompanied by a partial loss of its native secondary structure. With additional thermal stress in the presence of DMSO, a further decrease in Lys activity and native secondary structure was observed [36,38,41]. However, this decrease was much smaller than that in the absence of DMSO. Thus, DMSO can exert both destabilizing and stabilizing effects on the structure of Lys depending on solvent composition and thermal conditions [36,37,38,40,41]. This dual influence may favor partially unfolded states that are competent for aggregation under appropriate conditions [34,35,36,37,38,40,41,42].

**Figure 2:**
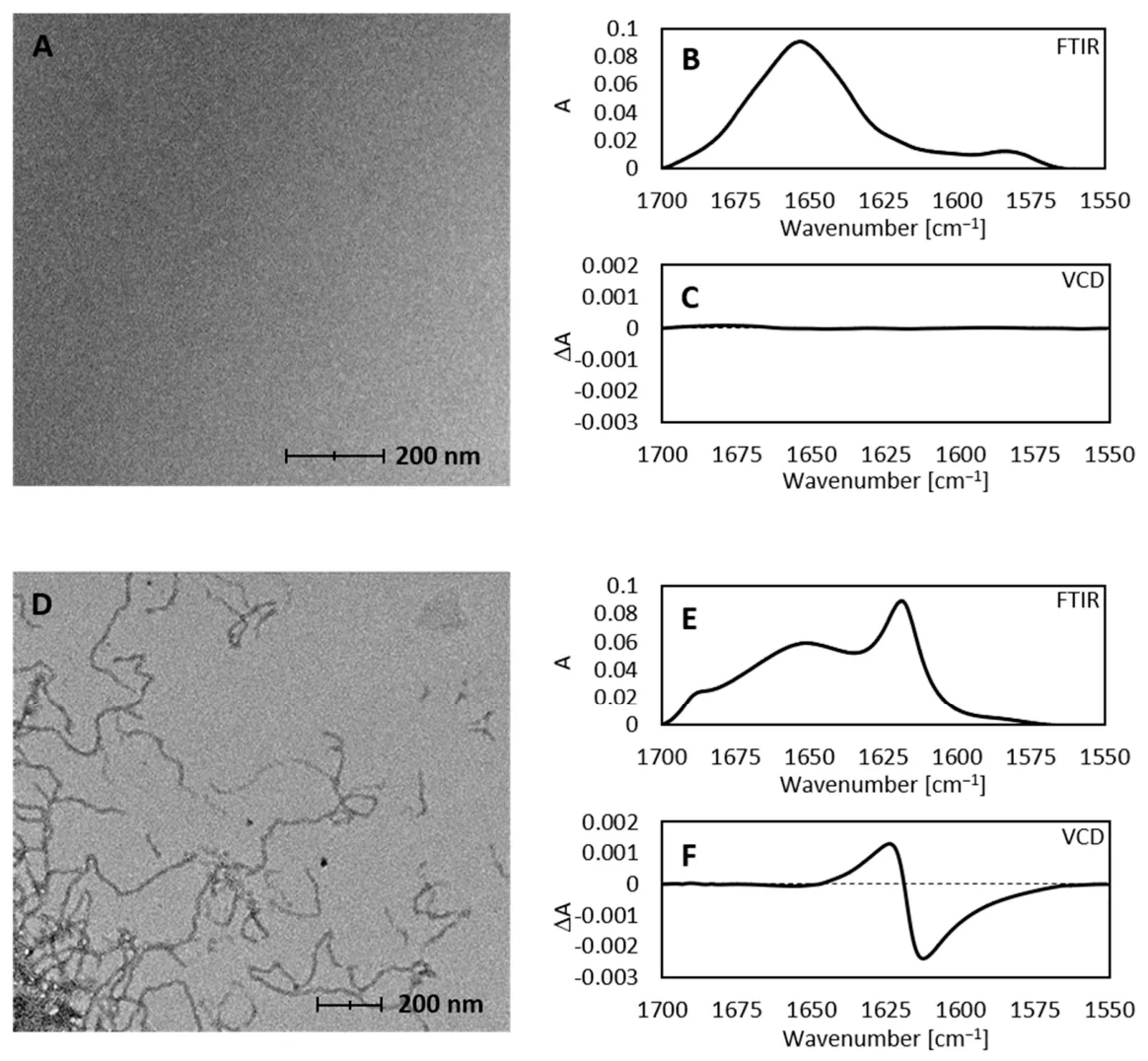
Transmission electron microscopy (TEM) images of Lys in the native form (**A**) and fibrillar aggregates of Lys proteins (**D**). The fourier transform infrared (FTIR) (**B**) and vibrational circular dichroism (VCD) (**C**) spectra of Lys in native form. The FTIR (**E**) and VCD (**F**) spectra of Lys fibrillar aggregates.

Figure 2 presents TEM images, FTIR spectra, and VCD spectra for Lys (120 mg/mL) in a DMSO-D_2_O mixture (x_DMSO_ = 0.30), comparing samples without and with thermal treatment. These results illustrate the unfolding and assembly processes of Lys under the experimental conditions used in this study. Dissolving Lys in D_2_O with x_DMSO_ = 0.30 at room temperature (without heating) leads to a partial loss of the ordered conformations characteristic of native Lys. In the FTIR spectrum (Figure 2B), the amide I band remains centered around 1651 cm^−1^, but its intensity is slightly reduced, indicating that the presence of DMSO induces some conversion of the α-helical structure (the dominant conformation in native Lys) into more disordered structures such as turns and bends [23,25,42,43,44]. Consistently, the TEM image (Figure 2A) shows that Lys treated with the DMSO-D_2_O mixture (x_DMSO_ = 0.30) at room temperature does not form any aggregated structures.

In contrast, when the Lys solution in the DMSO-D_2_O mixture (x_DMSO_ = 0.30) is thermalized at 70 °C for 30 min and then slowly cooled to room temperature, the sample forms a transparent gel containing fibrillar aggregates (Figure 2D). The FTIR spectrum of this gel (Figure 2E) shows significant changes in the secondary structure associated with fibril formation. Notably, the intensity of the amide I band around 1650 cm^−1^ (α-helix) is greatly diminished, indicating an extensive loss of native helical structure upon heating. Meanwhile, two new bands appear at approximately 1685 cm^−1^ and 1615 cm^−1^, which are indicative of intermolecular β-sheet structures. The emergence of these β-sheet bands confirms that partially unfolded Lys molecules have aggregated into cross-β fibrillar structures. Upon cooling, these β-sheet-rich structures become more stable and facilitate the formation of fibrils [18,22]. The VCD spectrum of the thermally treated sample (Figure 2F) displays a strong couplet with a positive peak at 1624 cm^−1^ and a negative peak at 1613 cm^−1^. According to the literature [18,20], the enhancement of VCD signal intensity is a signature of fibril formation and growth, and this specific (+/−) sign pattern is characteristic of Lys fibrils with left-handed supramolecular chirality (often referred to as the “normal” fibril form) [28].

**Figure 3:**
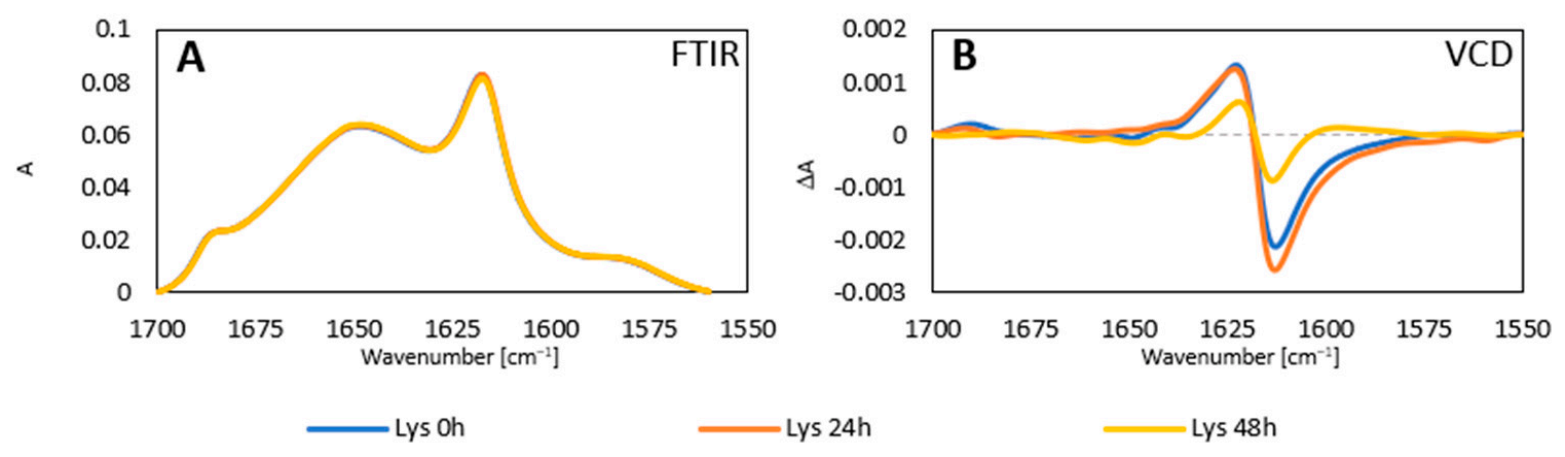
FTIR (**A**) and VCD (**B**) spectra of Lys fibrillar aggregates formed in a DMSO-rich water solution under thermal treatment with different incubation times (0 h, 24 h and 48 h) at room temperature.

Temperature-dependent secondary-structure reorganization and amyloid formation of Lys are also documented in more recent studies [28,34,35]. In the specific DMSO-rich system considered here, Sassi et al. [40,42] found that at temperatures above 65 °C, pre-formed Lys fibrils can begin to dissociate. The experiments were performed using a high protein concentration (120 mg/mL Lys), a high DMSO content x_DMSO_ = 0.30 and a 70 °C heating step. Under these conditions, Lys was observed to readily self-aggregate into fibrils (accompanied by misfolding, as shown in Figure 2), with the fibrils subsequently undergoing a gradual, time-dependent partial dissociation upon standing at room temperature (Figure 3). In particular, after 48 h at room temperature, the intensity of the fibril-specific VCD signals decreased markedly (Figure 3B), indicating that some of the aggregates had partially dissociated over time. Importantly, this dissociation process was not accompanied by any significant secondary-structure changes in the protein, as evidenced by the essentially unchanged amide I profiles in the FTIR spectra (Figure 3A).

### 2.2. Structural Polymorphism of Lysozyme in the Presence of Caffeic Acid

**Figure 4:**
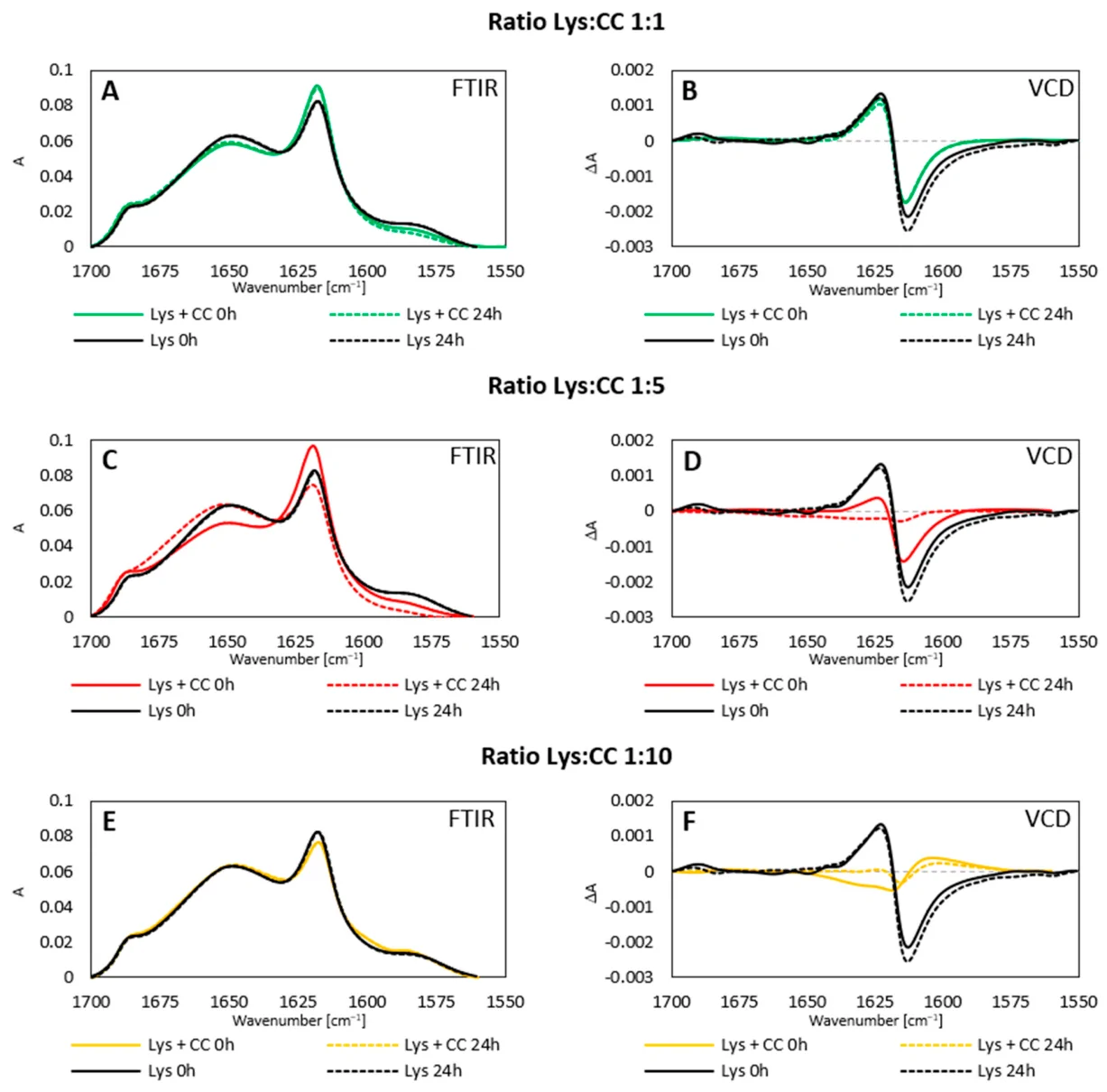
FTIR and VCD spectra of Lys fibrillar aggregates formed in the presence of CC at different Lys to polyphenol molar ratios in a DMSO-rich aqueous solution: (**A**) FTIR spectra for Lys:CC 1:1; (**B**) VCD spectra for Lys:CC 1:1; (**C**) FTIR spectra for Lys:CC 1:5; (**D**) VCD spectra for Lys:CC 1:5; (**E**) FTIR spectra for Lys:CC 1:10; (**F**) VCD spectra for Lys:CC 1:10. Spectra were recorded after thermal treatment and subsequent incubation at room temperature for 0 h and 24 h. Corresponding Lys samples without CC are shown for comparison.

The effect of each of the three phenolic compounds on the structural polymorphism of Lys fibrils was investigated next. Figure 4 shows FTIR and VCD spectra for Lys samples prepared with different concentrations of CC (Lys:CC molar ratios of 1:1, 1:5, and 1:10). In all cases, the thermal treatment of Lys in the DMSO-D_2_O mixture (x_DMSO_ = 0.30) led to similar changes in the FTIR spectra, regardless of CC presence (Figure 4A,C,E): namely, a partial loss of native α-helix structure and an increase in β-sheet content, consistent with fibril formation, just as observed for pure Lys. Thus, the temperature-induced secondary-structure rearrangements accompanying fibrillogenesis were largely unaffected by the presence of CC molecules at these concentrations.

Despite the FTIR showing no major differences, the VCD spectra revealed changes in fibril formation as a function of CC concentration (Figure 4B,D,F). Since an enhancement of the VCD signal is associated with fibril formation and can be used to monitor the development of fibrillar assemblies under comparable experimental conditions [28,30,31,32,33], the VCD intensity changes were used to assess the extent of fibril formation or fibril dissociation in the CC-treated samples. At the lowest CC level (Lys:CC 1:1), the VCD spectrum (Figure 4B) was very similar to that of pure Lys fibrils, suggesting that 1 equiv of CC had little to no effect on Lys fibril formation. However, at higher CC concentrations, clear effects on fibrillogenesis were observed. In the Lys:CC 1:5 sample, Lys did form fibrils with the characteristic left-handed supramolecular structure (indicated by the +1621/−1616 cm^−1^ VCD couplet), but the presence of CC reduced the extent of fibril formation: the VCD couplet intensity was about half of that for pure Lys (Figure 4D). These partially formed fibrillar aggregates were also less stable. After 24 h of incubation at room temperature, the VCD signal for the 1:5 sample nearly vanished, indicating that the CC had induced an almost complete dissociation of the fibrils over time. An even more pronounced inhibition was observed at the highest CC concentration (Lys:CC 1:10). In this case, virtually no fibrillar VCD signal was detected even at 0 h, and none appeared after 24 h (Figure 4F), implying that 10 equivalents of CC entirely prevented Lys from aggregating into detectable fibrils.

It should be mentioned that there is a difference in the FTIR signals between the Lys:CC 1:10 sample and the other two samples (Lys:CC 1:1 and Lys:CC 1:5) (see Figure 4A,C,E). This observation likely indicates that the modification of β-sheet structures in Lys by CC is a concentration-dependent process. Specifically, at the highest CC concentration, we observe a reduction in the intensity of the amide I band with a maximum at ~1615 cm^−1^, which is characteristic of β-sheet structures. This demonstrates that such a high concentration of CC effectively reduces the proportion of β-sheets in the studied protein relative to the amount present in the control protein.

**Figure 5:**
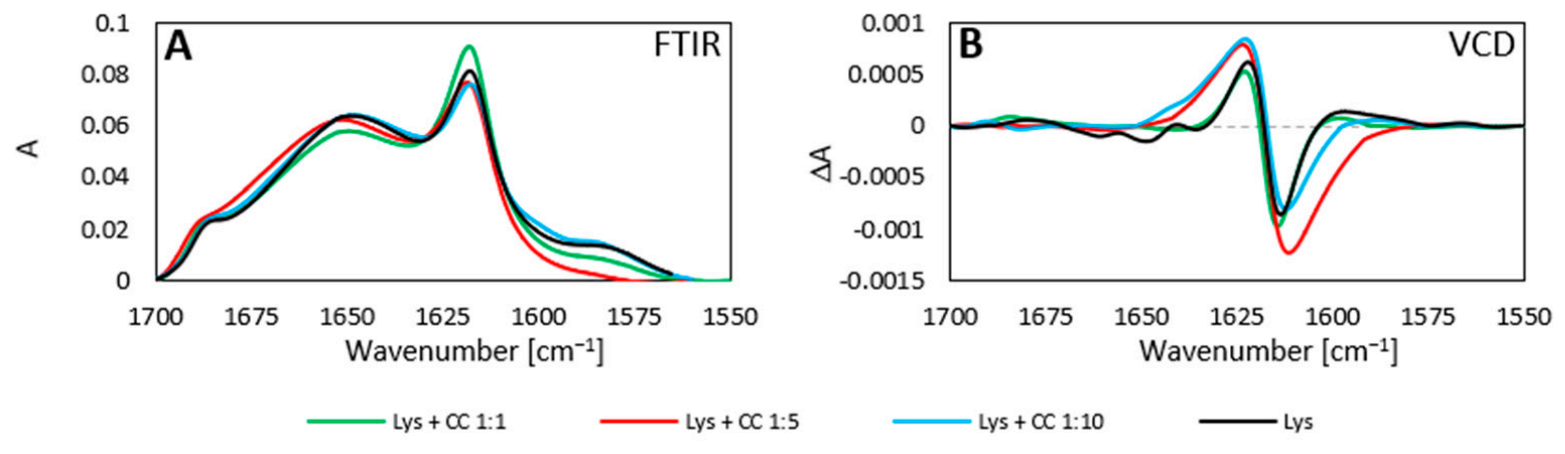
FTIR (**A**) and VCD (**B**) spectra of Lys fibrillar aggregates formed in the presence of CC molecules in different Lys to CC molar ratios (1:1, 1:5 and 1:10) in a DMSO-rich water solution under thermal treatment and then incubated at room temperature for 48 h.

As noted above, the unique experimental conditions used in this study enabled the observation of not only the formation of Lys fibrils but also their slow dissociation over time. Figure 5 illustrates the effect of CC on the time-dependent dissociation of Lys fibrils. Generally, the presence of CC did not significantly alter the extent of fibril dissociation during prolonged incubation at room temperature. In all the CC-containing samples (1:1, 1:5, 1:10), the VCD intensity after 48 h decreased to a similarly low level as in the pure Lys sample (compare Figure 5B with Figure 3B), indicating that fibrils eventually dissociate to a similar degree with or without CC under these conditions.

### 2.3. Structural Polymorphism of Lysozyme in the Presence of Quercetin

**Figure 6:**
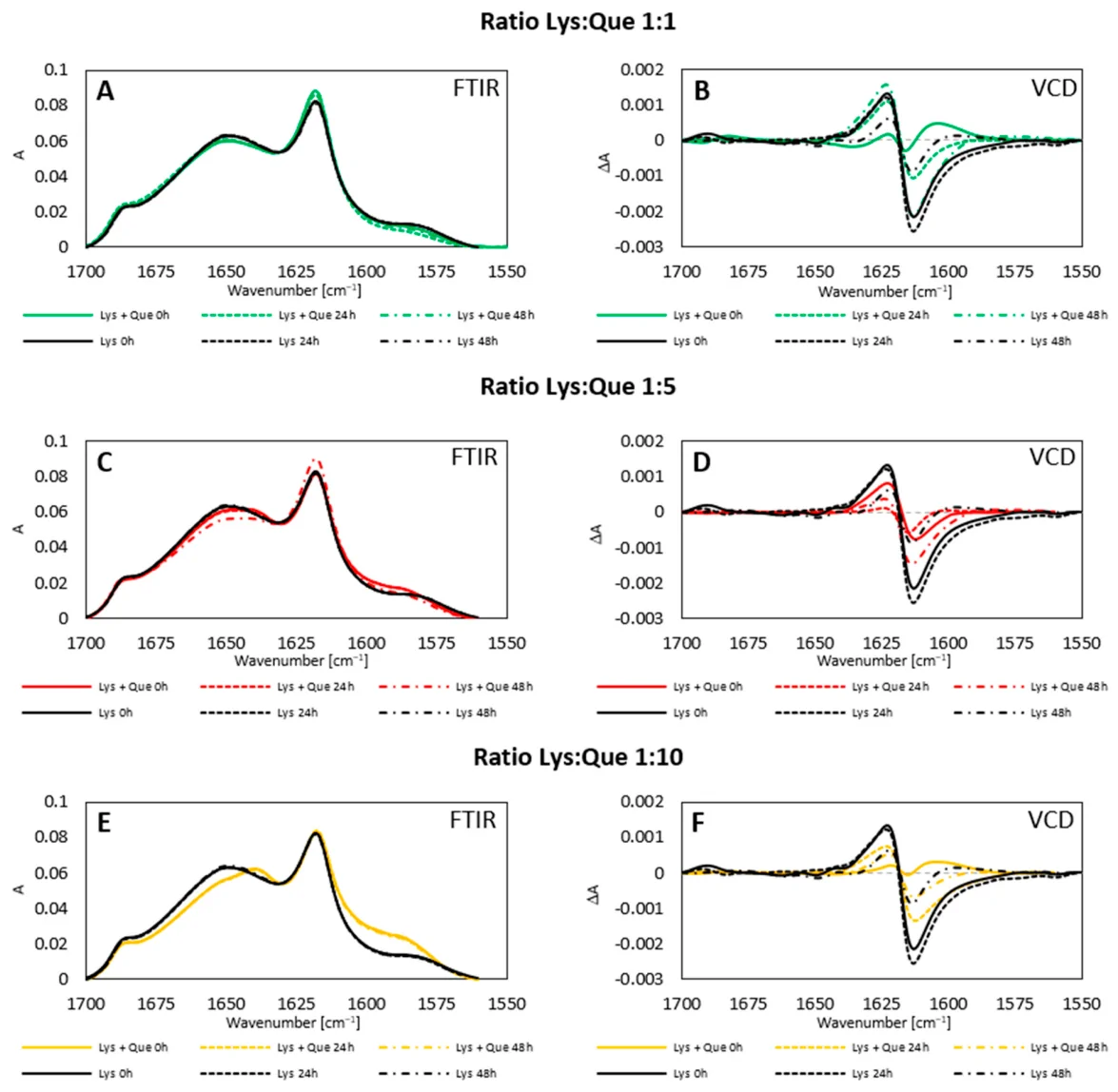
FTIR and VCD spectra of Lys fibrillar aggregates formed in the presence of Que at different Lys to polyphenol molar ratios in a DMSO-rich aqueous solution: (**A**) FTIR spectra for Lys:Que 1:1; (**B**) VCD spectra for Lys:Que 1:1; (**C**) FTIR spectra for Lys:Que 1:5; (**D**) VCD spectra for Lys:Que 1:5; (**E**) FTIR spectra for Lys:Que 1:10; (**F**) VCD spectra for Lys:Que 1:10. Spectra were recorded after thermal treatment and subsequent incubation at room temperature for 0 h, 24 h, and 48 h. Corresponding Lys samples without Que are shown for comparison.

The effect of Que on Lys fibrillogenesis was examined analogously. Figure 6 shows FTIR and VCD spectra for Lys samples prepared with Que at Lys:Que ratios of 1:1, 1:5, and 1:10. As with the CC-treated samples, the presence of Que had little or no impact on the secondary-structure changes induced by the thermal treatment. In all Que-containing samples, the FTIR spectra (Figure 6A,C,E) show the same features as seen for pure Lys: a decrease in the 1650 cm^−1^ band (loss of α-helix) and growth of β-sheet bands, indicating that Que did not prevent the initial unfolding and β-sheet formation.

However, Que did modulate the extent of fibril formation, as evidenced by the VCD results. All the Que-treated samples showed a marked inhibition of Lys fibril formation at 0 h (immediately after cooling). The VCD signals were significantly lower in intensity compared to pure Lys, indicating fewer or smaller fibrils (Figure 6B,D,F). This inhibitory effect of Que on fibrillogenesis, however, diminished over time. After 24 h at room temperature, the VCD signals characteristic of left-handed fibrils became more intense in all Que-treated samples (compare the 0 h and 24 h spectra in Figure 6B,D,F), suggesting that some fibril growth occurred during the incubation period despite the presence of Que. Additionally, Que had only a minor influence on the long-term dissociation of fibrils. After 48 h, the VCD spectra of the 1:5 and 1:10 Que samples were very similar to those of pure Lys (Figure 6D,F), indicating that these samples ended up with a comparable level of residual fibrils. Interestingly, for the lowest Que concentration (Lys:Que 1:1), the 48 h VCD signal was actually more intense than that of pure Lys at 48 h (Figure 6B). This suggests that a sub-stoichiometric amount of Que (1:1) might slow the fibril dissociation relative to pure Lys or even promote some late-stage fibril assembly, although the exact reason for this increase is not yet clear. In agreement with our finding that CC and Que exert distinct effects on fibrillogenesis, López et al. [18] demonstrated that under physiological conditions, CC clearly inhibited human Lys fibrillation across multiple assays (ThT fluorescence, 8-anilinonaphthalene-1-sulfonic acid (ANS) fluorescence, circular dichroism (CD) spectroscopy, and scanning electron microscopy (SEM)). Conversely, Que produced a highly assay-dependent response, characterized by distinct ANS and CD signatures but little change in ThT fluorescence.

### 2.4. Structural Polymorphism of Lysozyme in the Presence of Resveratrol

**Figure 7:**
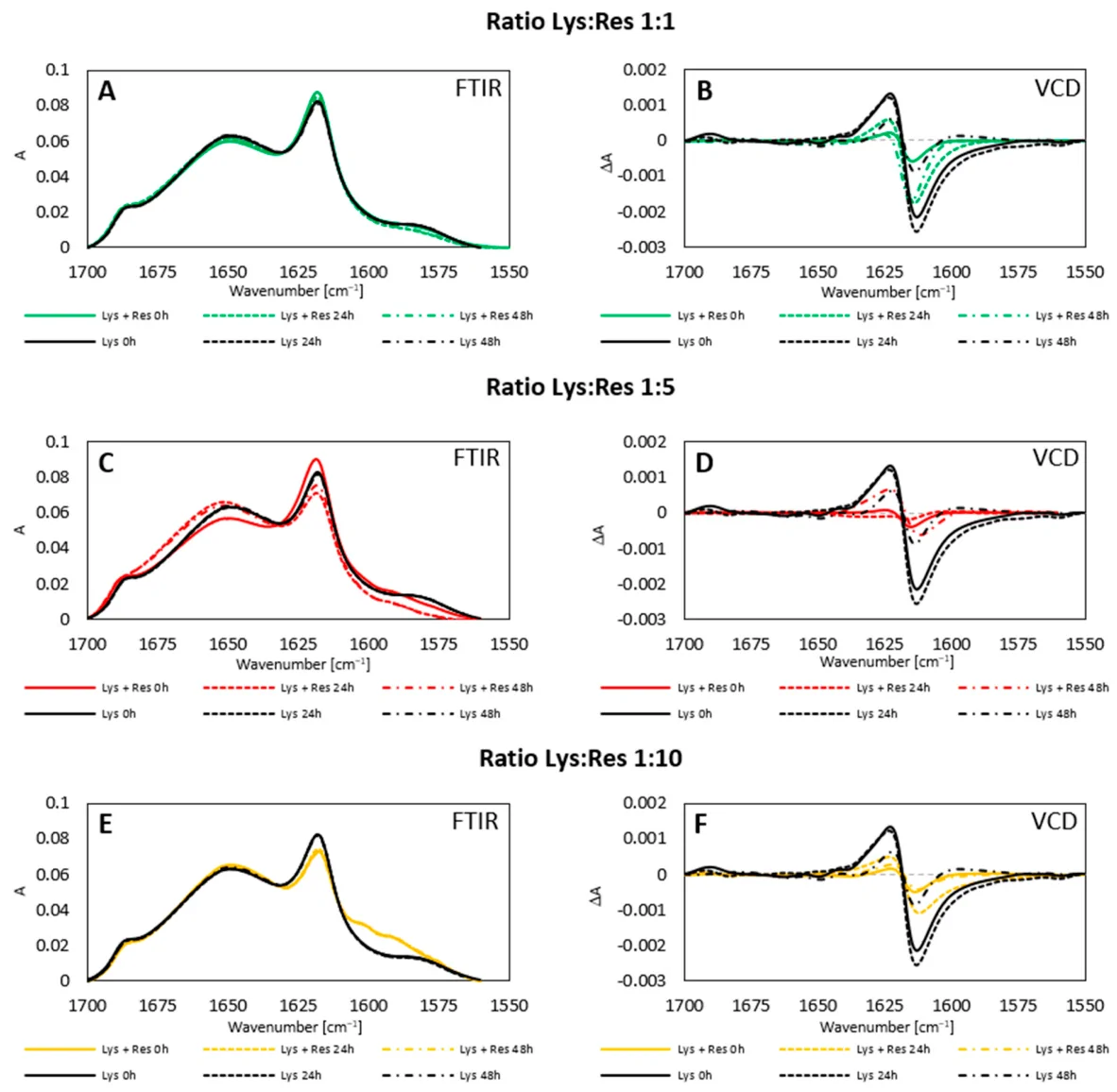
FTIR and VCD spectra of Lys fibrillar aggregates formed in the presence of Res at different Lys to polyphenol molar ratios in a DMSO-rich aqueous solution: (**A**) FTIR spectra for Lys:Res 1:1; (**B**) VCD spectra for Lys:Res 1:1; (**C**) FTIR spectra for Lys:Res 1:5; (**D**) VCD spectra for Lys:Res 1:5; (**E**) FTIR spectra for Lys:Res 1:10; (**F**) VCD spectra for Lys:Res 1:10. Spectra were recorded after thermal treatment and subsequent incubation at room temperature for 0 h, 24 h, and 48 h. Corresponding Lys samples without Res are shown for comparison.

Figure 7 shows analogous data for Lys samples treated with Res at Lys:Res ratios of 1:1, 1:5, and 1:10. The FTIR spectra (Figure 7A,C,E) were very similar for all of these samples, indicating that Res, like CC and Que, did not significantly alter the secondary-structure evolution during the thermal induction of fibrils. In other words, even in the presence of Res, Lys underwent the characteristic partial unfolding and increased β-sheet content that favor fibril formation.

Notably, however, fibril assembly was strongly suppressed in the Res-treated samples. Even though the Lys molecules adopted secondary structures conducive to fibrillation (as confirmed by FTIR), they largely failed to aggregate into fibrils when Res was present. Even at the lowest Res concentration (Lys:Res 1:1), the VCD spectrum showed only a very weak fibrillar signal, implying that Res substantially reduced the formation of amyloid fibrils. Among the three phenols tested, Res proved to be the most effective inhibitor of fibril formation: it produced the largest reduction in fibril-related VCD intensity (compare Figure 7B with Figure 4B and Figure 6B). Although the inhibitory effect of Res did weaken over time (as some fibrils gradually formed during incubation), it diminished to the smallest extent compared to the cases of CC and Que. The inhibitory effect of Res on Lys fibrillation is further supported by independent, orthogonal studies. For instance, Shariatizi et al. [45] demonstrated a concentration-dependent inhibition of HEWL fibrillation; their ThT assay results, corroborated by TEM and CD, revealed reduced fibril formation and a delayed transition into β-sheet-rich structures. Similarly, Zaidi and Bhat [46] reported a concentration-dependent inhibition of human Lys fibrillation, where ThT findings—validated by TEM and DLS—indicated that the pathway was redirected toward morphologically distinct off-pathway aggregates.

### 2.5. Structural Polymorphism of Lysozyme in the Presence of Polyphenolic Mixtures

**Figure 8:**
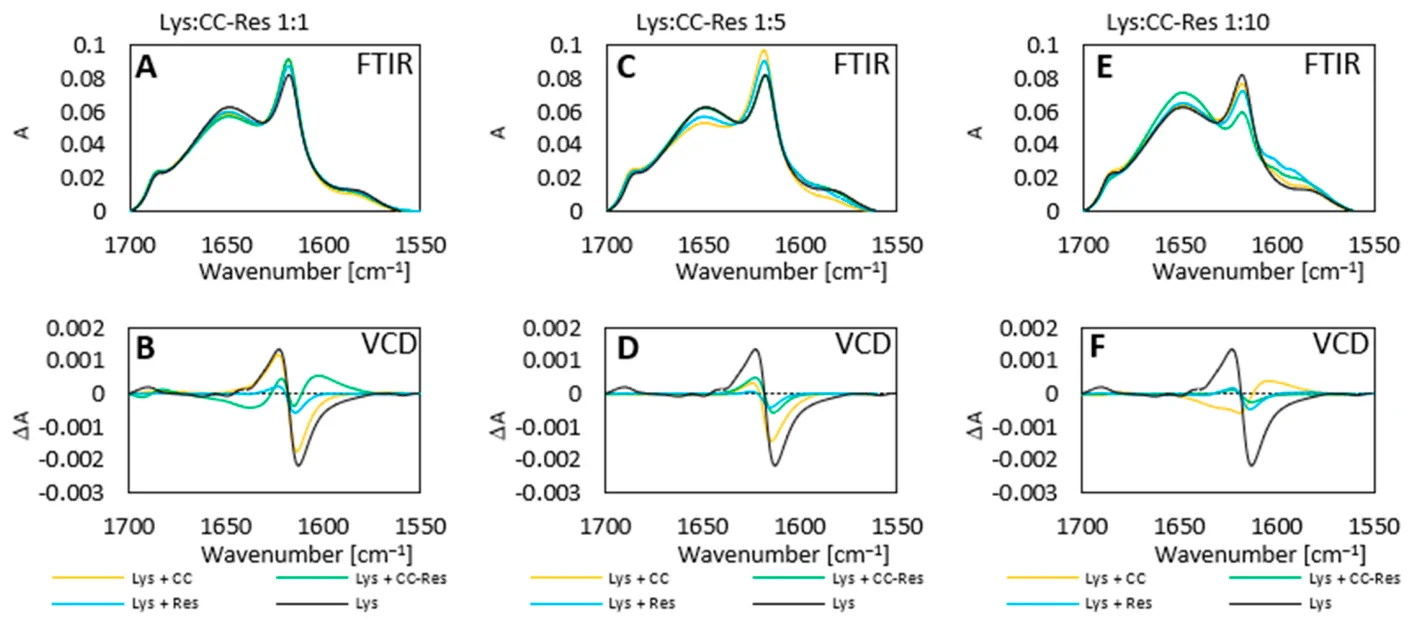
FTIR and VCD spectra of Lys fibrillar aggregates formed in the presence of an equimolar mixture of CC and Res at different Lys to polyphenols molar ratios in a DMSO-rich aqueous solution: (**A**) FTIR spectra for a Lys:CC-Res ratio of 1:1; (**B**) VCD spectra for a Lys:CC-Res ratio of 1:1; (**C**) FTIR spectra for a Lys:CC-Res ratio of 1:5; (**D**) VCD spectra for a Lys:CC-Res ratio of 1:5; (**E**) FTIR spectra for a Lys:CC-Res ratio of 1:10; (**F**) VCD spectra for a Lys ratio of 1:10. Spectra were recorded immediately after thermal treatment, without subsequent incubation at room temperature (0 h).

The influence of binary and tertiary mixtures of the phenolic compounds on Lys fibrillation was also examined. Figure 8 presents data for an equimolar binary mixture of CC and Res at overall Lys:phenol ratios of 1:1, 1:5, and 1:10. Res alone is a very effective inhibitor of Lys fibril formation (even at 1:1, as shown in Figure 7), whereas CC alone at 1:1 has almost no inhibitory effect (Figure 4). Interestingly, when half of the less-active CC was replaced by the highly active Res, the mixture still inhibited fibrillation very effectively. In the Lys:CC+Res 1:1 sample, fibril formation was almost completely suppressed—the FTIR showed only minimal β-sheet aggregation, and the VCD signal was extremely weak (Figure 8A,B)—essentially as effective as Res alone. Similarly, the Lys:CC+Res 1:5 mixture markedly reduced fibril formation compared to either phenol individually (Figure 8C,D). The combined effect of CC and Res in these samples is greater than the sum of their separate effects, suggesting a synergistic interaction between the two compounds. At the highest total phenolic content (Lys:phenols 1:10), fibril formation was completely inhibited in all cases (with CC alone, Res alone, or the CC + Res mixture), as evidenced by the absence of β-sheet peaks in FTIR and the lack of a VCD signal (Figure 8E,F). It is worth noting that in the Lys:CC+Res 1:10 sample, the inhibition of fibrillation was accompanied by a noticeable shift in the FTIR amide I profile: the intensity of the aggregated β-sheet band at 1615 cm^−1^ was strongly reduced, while the band at 1648 cm^−1^ (associated with disordered structures) was increased (Figure 8E). This suggests that when fibril formation is completely blocked, the partially unfolded proteins remain in a more disordered state rather than converting into β-sheet aggregates.

**Figure 9:**
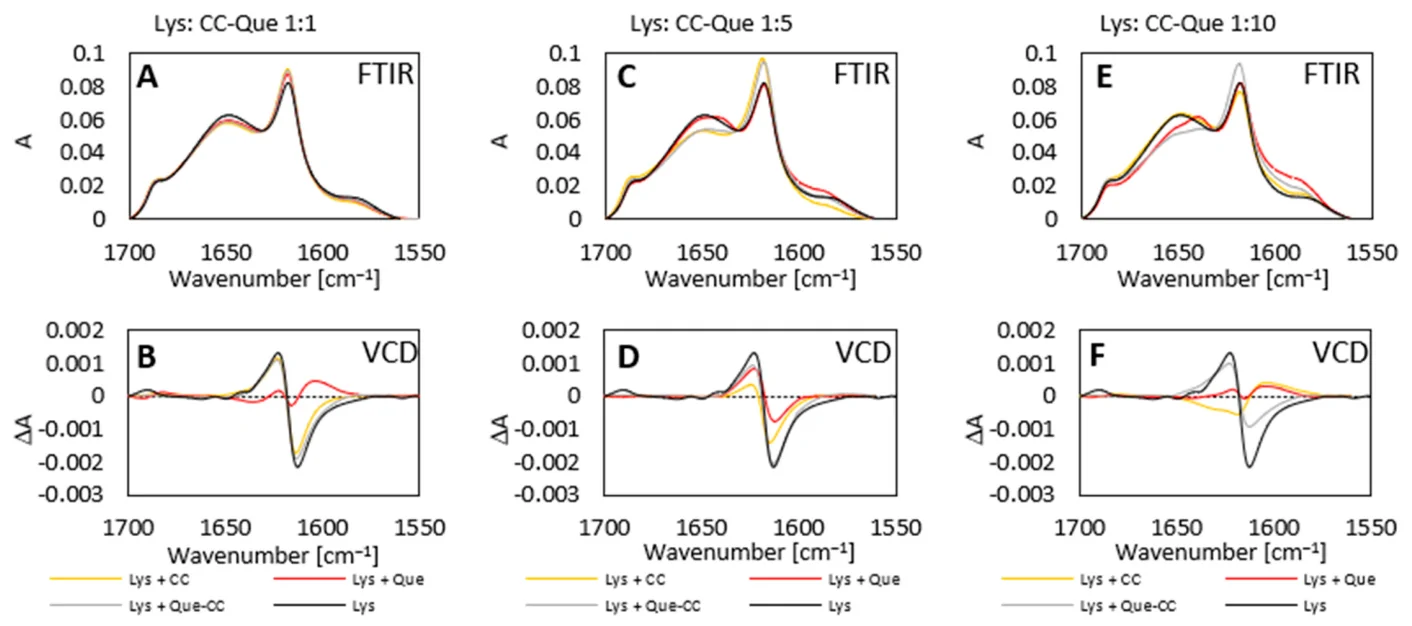
FTIR and VCD spectra of Lys fibrillar aggregates formed in the presence of an equimolar mixture of CC and Que at different Lys to polyphenols molar ratios in a DMSO-rich aqueous solution: (**A**) FTIR spectra for a Lys:CC-Que ratio of 1:1; (**B**) VCD spectra for a Lys:CC-Que ratio of 1:1; (**C**) FTIR spectra for a Lys:CC-Que ratio of 1:5; (**D**) VCD spectra for a Lys:CC-Que ratio of 1:5; (**E**) FTIR spectra for a Lys:CC-Que ratio of 1:10; (**F**) VCD spectra for a Lys:CC-Que ratio of 1:10. Spectra were recorded immediately after thermal treatment, without subsequent incubation at room temperature (0 h).

Figure 9 shows the results for equimolar mixtures of CC and Que at Lys:phenol ratios of 1:1, 1:5, and 1:10. As described above, Que alone (especially at 1:1) initially inhibits fibril formation (Figure 6), whereas CC alone has little effect at 1:1 (Figure 4). When Que was combined with CC in an equimolar mixture, we observed an antagonistic effect. In all CC+Que mixture samples, the inhibitory action of Que was nullified by the presence of CC, resulting in significant fibril formation. For example, in the Lys:CC+Que 1:1 sample, the VCD spectrum at 0 h showed a strong fibril signal (Figure 9B), in stark contrast to the weak signal seen with Que alone (Figure 6B). The FTIR data support this: the CC+Que mixtures exhibited increased β-sheet content (stronger 1615 cm^−1^ band) and decreased disordered structure (weaker 1648 cm^−1^ band) relative to samples with Que alone (Figure 9A,C,E). Thus, adding CC effectively counteracted the fibril inhibition by Que, promoting fibril formation instead. This antagonistic interaction persisted at higher phenol concentrations as well, although the overall fibril yield was lower at 1:5 and 1:10 due to Que’s presence (Figure 9D,F).

**Figure 10:**
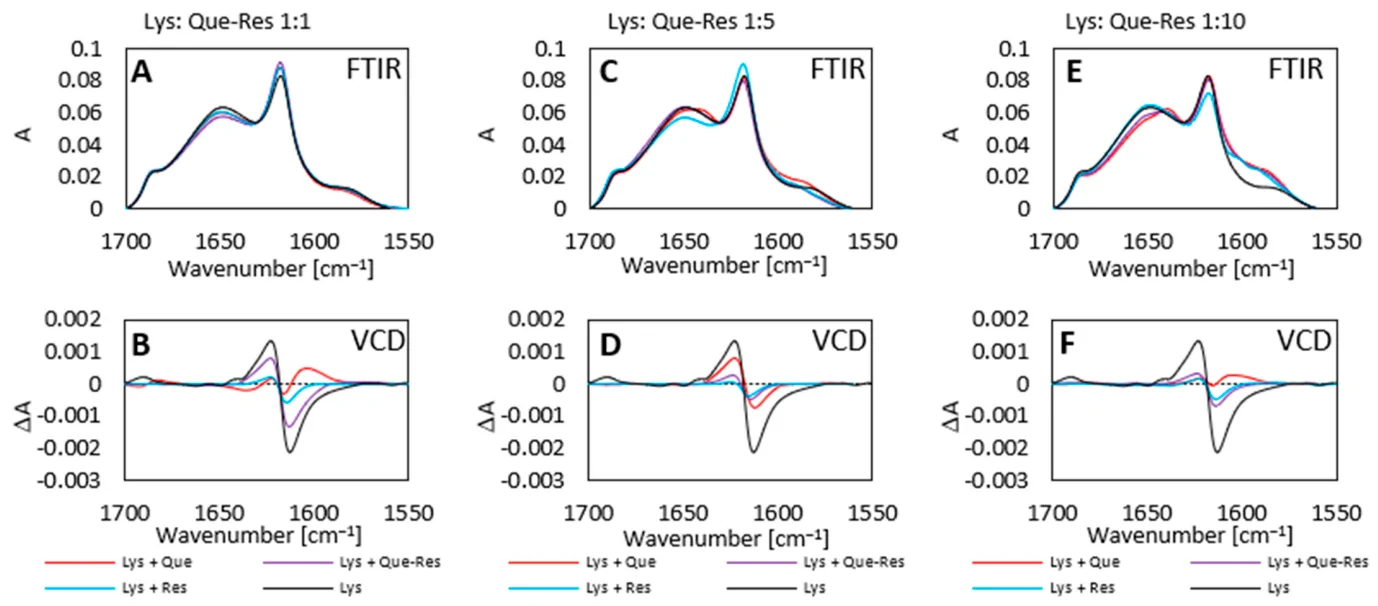
FTIR and VCD spectra of Lys fibrillar aggregates formed in the presence of an equimolar mixture of Que and Res at different Lys to polyphenols molar ratios in a DMSO-rich aqueous solution: (**A**) FTIR spectra for a Lys:Que-Res molar ratio of 1:1; (**B**) VCD spectra for a Lys:Que-Res molar ratio of 1:1; (**C**) FTIR spectra for a Lys:Que-Res molar ratio of 1:5; (**D**) VCD spectra for a Lys:Que-Res molar ratio of 1:5; (**E**) FTIR spectra for a Lys:Que-Res molar ratio of 1:10; (**F**) VCD spectra for a Lys:Que-Res molar ratio of 1:10. Spectra were recorded immediately after thermal treatment, without subsequent incubation at room temperature (0 h).

Figure 10 explores mixtures of Que and Res (equimolar) at Lys:phenol ratios of 1:1, 1:5, and 1:10. Both Que and Res individually inhibit fibril formation, with Res being more potent. When combined, their effects were somewhat antagonistic, particularly at the lowest total concentration. In the Lys:Que+Res 1:1 sample, the VCD signal for left-handed fibrils was relatively high (Figure 10B), indicating that significant fibril formation still occurred—more than one would expect if Res’s strong inhibition was fully effective. Thus, at 1:1, the presence of Que appeared to diminish Res’s inhibitory impact (or vice versa), resulting in more fibrils than with Res alone (suggesting a partial antagonism). At higher phenol contents (1:5 and 1:10), fibril formation was strongly suppressed (Figure 10D,F), as both compounds were abundant, but even in these cases, the combined inhibition was slightly less than what Res alone achieved at the same total concentration (i.e., the mixtures showed a minor antagonistic tendency).

**Figure 11:**
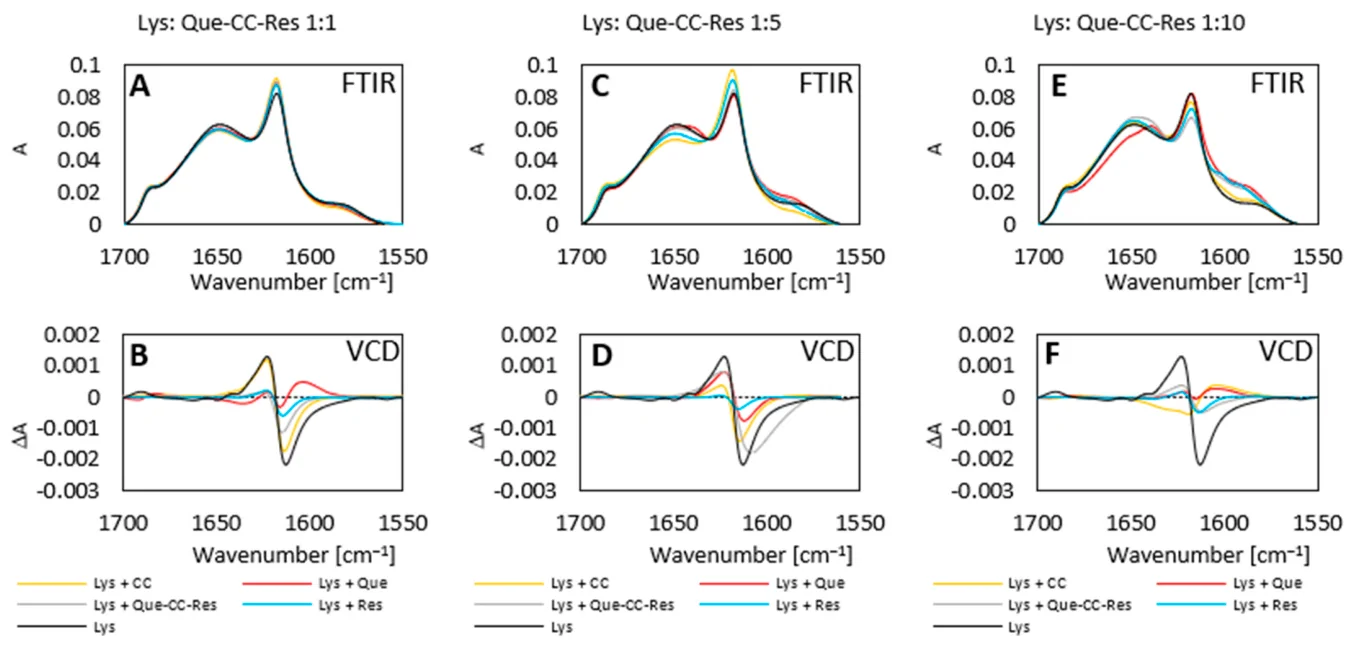
FTIR and VCD spectra of Lys fibrillar aggregates formed in the presence of a ternary mixture of Que, CC, and Res at different Lys to pholyphenols molar ratios in a DMSO-rich aqueous solution: (**A**) FTIR spectra for a Lys:Que-CC-Res ratio of 1:1; (**B**) VCD spectra for a Lys:Que-CC-Res ratio of 1:1; (**C**) FTIR spectra for a Lys:Que-CC-Res ratio of 1:5; (**D**) VCD spectra for a Lys:Que-CC-Res ratio of 1:5; (**E**) FTIR spectra for a Lys:Que-CC-Res ratio of 1:10; (**F**) VCD spectra for a Lys:Que-CC-Res ratio of 1:10. Spectra were recorded immediately after thermal treatment, without subsequent incubation at room temperature (0 h).

Finally, testing was performed using a tertiary mixture containing Que, CC, and Res in equimolar amounts (Que + CC + Res), at the same overall phenol ratios (1:1, 1:5, 1:10). The effects of this complex mixture on Lys fibrillation are summarized in Figure 11. The outcome with all three phenols together was not straightforward: depending on the concentration, the mixture’s behavior could be either synergistic or antagonistic. At the lowest concentration (Lys:phenols (1:1)), the presence of all three phenols did not inhibit fibril formation as effectively as Res or the CC+Res pair alone, indicating diminished anti-fibrillar activity (an antagonistic outcome). At higher concentrations, however, the mixture still managed to greatly reduce fibril formation, in some cases matching the efficacy of the best binary combination. In the 1:5 and 1:10 tertiary samples, either nearly complete inhibition of fibrils or a level of inhibition comparable to the best binary mixtures was observed, suggesting some synergistic cooperation at those higher doses. In summary, the Que–CC–Res tertiary mixture did not have a clearly defined effect; its components can act synergistically or antagonistically depending on their relative amounts, making its influence on fibrillation more complex to interpret (Figure 11B,D,F).

## 3. Materials and Methods

### 3.1. Materials

Resveratrol (Res, purity > 99.2%), quercetin (Que, purity > 95%), and caffeic acid (CC, purity > 95%) were obtained from Sigma-Aldrich Chemie GmbH (Taufkirchen, Germany). Hen egg-white lysozyme (Lys, molecular weight ~14.3 kDa, purity > 90%) was from Sigma-Aldrich Chemie GmbH (Taufkirchen, Germany). Dimethyl sulfoxide (DMSO, purity > 99.7%) was from Chempur (Piekary Śląskie, Poland), and deuterium oxide (D_2_O, purity > 99.9%) was from Sigma-Aldrich Chemie GmbH (Taufkirchen, Germany). All chemicals were used as received without further purification.

### 3.2. Preparation of Lysozyme Fibrils

Lys fibrils were prepared following a modified version of the method reported in [18]. A 15 mg portion of Lys was dissolved in 46.6 µL of D_2_O. As required, an appropriate volume of a phenolic stock solution (Res, Que, or CC) was added to achieve the desired Lys-to-total polyphenol molar ratios of 1:1, 1:5, or 1:10. DMSO was then added to adjust the cosolvent mole fraction to x_DMSO_ = 0.30. The total sample volume was 125 µL, and the final Lys concentration was 120 mg/mL (approximately 8.4 mM). The samples were incubated (thermalized) at 70 °C for 30 min, followed by gradual cooling to room temperature (≈22 °C) using a Julabo F25-MC water bath with automatic temperature control (JULABO Labortechnik GmbH; Seelbach, Germany). After this treatment, each sample was divided for measurements at different time points: FTIR and VCD spectra were recorded immediately after preparation (0 h) and then the samples were stored at room temperature and measured again after 24 h and 48 h of incubation.

### 3.3. Preparation of Polyphenol Stock Solutions

Stock solutions of the phenolic compounds were prepared in DMSO. Specifically, 51.2 mg of Res was dissolved in 250 µL of DMSO (≈0.9 M stock); 85.0 mg of Que in 1500 µL DMSO (≈0.19 M); and 25.0 mg of CC in 100 µL DMSO (≈1.4 M). These stock solutions were used to obtain samples with the specified Lys:phenol molar ratios (using the total moles of phenols in the case of mixtures).

### 3.4. FTIR and VCD Measurements

For spectroscopic measurements, samples were placed in a demountable cell with two CaF_2_ windows separated by a 24 µm Teflon spacer. FTIR and VCD spectra were recorded at room temperature (~22 °C) using a Nicolet iS50 FTIR spectrometer (Thermo Scientific; Waltham, MA, USA) equipped with an iS50 FT-VCD module (Thermo Scientific; Waltham, MA, USA), PEM-100 photoelastic modulator (Hinds Instruments, Inc.; Hillsboro, OR, USA), and synchronous sampling demodulator (GWC Technologies; Madison, WI, USA). For each spectrum, 128 scans were co-added for FTIR (resolution 2 cm^−1^) and 400 scans for VCD (resolution 8 cm^−1^), over the range of 4000–400 cm^−1^. The VCD spectra are presented as the differential absorbance (ΔA) of left- and right-circularly polarized light. Following data acquisition, the spectra were baseline-corrected as needed and normalized to the integrated area of the amide I band in the FTIR spectrum. This normalization allows a direct comparison of relative VCD signal intensities between samples.

The lack of fibrils was proved by the flat VCD signal and the FTIR spectrum with the amide I band of the native α-helical/random coil structures; this situation is presented in Figure 2A–C. Dissociation of fibrils, during which fibrils lose their supramolecular chirality, should manifest over time as a decrease in intensity of the VCD signal; as an example, see the time-dependent results presented in Figure 4. The native Lys is composed exclusively of L-amino acids and the formation of a perfectly racemic mixture of left- and right-handed supramolecular fibrils (manifested as the flat VCD signal) is highly improbable in this biological protein.

## 4. Conclusions

FTIR and VCD spectroscopy were employed to study the ability of single phenolic compounds, as well as mixtures of these compounds, to modulate the formation of Lys fibrillar aggregates. All of the phenolic compounds used in this study (CC, Que, and Res) are established modulators of protein and peptide fibrillation, but their effects can range from inhibition to promotion depending on the compound, concentration, target protein, and experimental conditions [14,15,16,17,18,19,20]. Their effects have commonly been evaluated using ThT fluorescence together with complementary spectroscopic and microscopic methods [14,15,18,19,20]. Here, a comprehensive, systematic investigation was presented of how CC, Que, and Res affect Lys fibrillogenesis depending on the phenol concentration, the way they are combined, and the experimental conditions. VCD proved to be an excel-lent method with high sensitivity to the formation and growth of fibril-like aggregated structures [28,30,31,32,33]. Moreover, VCD enabled the determination of the supramolecular chirality of the aggregates, revealing whether the formed fibrils are left- or right-handed. To the best of available knowledge, this work provides the first evidence that Lys can form left-handed fibrils in the presence of each of these phenolic modulators. The polyphenols primarily strengthened or weakened the fibril, without changing the intrinsic left-handed twist of the aggregates. It is worth mentioning that differences in VCD intensity do not automatically represent differences in fibril concentration. The relationship between higher VCD intensity and a larger amount of fibrils is not necessarily linear, since VCD enhancement also depends on supramolecular order, fibril morphology, orientation, optical coupling, and scattering. For this reason, in this paper, we discuss changes in VCD intensity not in terms of fibril concentration, but in terms of the formation and growth of fibril aggregates (i.e., the transition from young filaments with low VCD intensity to mature fibrils with high VCD intensity).

Broadly, the effects of CC, Que, and Res observed under the experimental conditions used in this study are consistent with recent literature showing molecule- and condition-dependent modulations of Lys fibrillation by phenolic compounds [14,18,19,20]. In the present system, CC and Res were more effective at suppressing Lys fibrillation than Que. Furthermore, an equimolar mixture of CC and Res showed a strong synergistic effect in inhibiting Lys fibril formation, whereas a CC + Que mixture exhibited an antagonistic effect. This pattern is consistent with the system-specific findings of Gazova et al. [14]. Overall, the results highlight the utility of FTIR and VCD as label-free techniques for detecting and characterizing protein fibrils and their modulation by small molecules [23,25,31,32,33]. This approach can provide valuable insights for developing strategies aimed at preventing or disrupting pathological protein aggregation.

While our spectroscopic results primarily highlight the macroscopic structural transitions, the inhibition of Lys fibrillogenesis by these phenolic compounds can be also mediated by complex mechanistic factors at the molecular level, including hydrogen bonding, aromatic stacking, hydrophobic interactions, and specific preferential binding sites [14,18].

## Data Availability

The data are contained within the article.

## References

[1] Dobson C.M. (2001). The structural basis of protein folding and its links with human disease. Philos. Trans. Lond. B Biol. Sci..

[2] Khlistunova I., Biernat J., Wang Y.P., Pickhardt M., von Bergen M., Gazova Z., Mandelkow E., Mandelkow E.M. (2006). Inducible expression of Tau repeat domain in cell models of tauopathy: Aggregation is toxic to cells but can be reversed by inhibitor drugs. J. Biol. Chem..

[3] Baglioni S., Casamenti F., Bucciantini M., Luheshi L.M., Taddei N., Chiti F., Dobson C.M., Stefani M. (2006). Prefibrillar amyloid aggregates could be generic toxins in higher organisms. J. Neurosci..

[4] Pastor M.T., Serrano L., Esteras-Chopo A. (2008). Protein misfolding and β-amyloid formation. Protein Folding, Misfolding and Aggregation: Classical Themes and Novel Approaches.

[5] Ng B., Vowles J., Bertherat F., Abey A., Kilfeather P., Beccano-Kelly D., Stefana M.I., O’Brien D.P., Bengoa-Vergniory N., Carling P.J. (2024). Tau depletion in human neurons mitigates Aβ-driven toxicity. Mol. Psychiatry.

[6] Scheiblich H., Eikens F., Wischhof L., Opitz S., Jüngling K., Cserép C., Schmidt S.V., Lambertz J., Bellande T., Pósfai B. (2024). Microglia rescue neurons from aggregate-induced neuronal dysfunction and death through tunneling nanotubes. Neuron.

[7] Urayama A., Moreno-Gonzalez I., Morales-Scheihing D., Kharat V., Pritzkow S., Soto C. (2022). Preventive and therapeutic reduction of amyloid deposition and behavioral impairments in a model of Alzheimer’s disease by whole blood exchange. Mol. Psychiatry.

[8] Roberson E.D., Scearce-Levie K., Palop J.J., Yan F., Cheng I.H., Wu T., Gerstein H., Yu G.Q., Mucke L. (2007). Reducing endogenous tau ameliorates amyloid beta-induced deficits in an Alzheimer’s disease mouse model. Science.

[9] De Felice F.G., Ferreira S.T. (2002). Beta-amyloid production, aggregation and clearance as targets for therapy in Alzheimer’s disease. Cell. Mol. Neurobiol..

[10] Liu J., Wang Z., Liang W., Zhang Z., Deng Y., Chen X., Hou Z., Xie Y., Wang Q., Li Y. (2025). Microglial TMEM119 binds to amyloid-β to promote its clearance in an Aβ-depositing mouse model of Alzheimer’s disease. Immunity.

[11] Gazit E. (2002). A possible role for π-stacking in self-assembly of amyloid fibrils. FASEB J..

[12] Qi K., Qi H., Wang M., Ma X., Wang Y., Yao Q., Liu W., Zhao Y., Wang J., Wang Y. (2024). Chiral inversion induced by aromatic interactions in short peptide assembly. Nat. Commun..

[13] Jayawardena B.M., Azzi A., Jones C.E. (2024). Investigating the role of phenylalanine residues for amyloid formation of the neu-ropeptide neurokinin B. Biochem. Biophys. Res. Commun..

[14] Gazova Z., Siposova K., Kurin E., Mucaji P., Nagy M. (2013). Amyloid aggregation of lysozyme: The synergy study of red wine polyphenols. Proteins.

[15] Di Giovanni S., Eleuteri S., Paleologou K.E., Yin G., Zweckstetter M., Carrupt P.A., Lashuel H.A. (2010). Entacapone and tolcapone, two catechol O-methyltransferase inhibitors, block fibril formation of α-synuclein and β-amyloid and protect against amyloid-induced toxicity. J. Biol. Chem..

[16] Kotormán M., Kelemen Z., Kasi P.B., Nemcsók J. (2018). Inhibition of the formation of amyloid-like fibrils using herbal extracts. Acta Biol. Hung..

[17] Cieślik-Boczula K., Trombik P. (2019). Resveratrol modulates fibrillogenesis in a poly-L-lysine peptide. Int. J. Biol. Macromol..

[18] López S., Rojo-Domínguez A., Nájera H. (2025). Impact of Phenolic Compounds on Fibrillogenesis of Human Lysozyme Under Physiological Conditions: Inhibition or Promotion?. ACS Omega.

[19] Shabnam, Bhat R. (2024). Flavones Suppress Aggregation and Amyloid Fibril Formation of Human Lysozyme under Macromolecular Crowding Conditions. Biochemistry.

[20] Gancar M., Kurin E., Bednarikova Z., Marek J., Mucaji P., Nagy M., Gazova Z. (2023). Green tea leaf constituents inhibit the formation of lysozyme amyloid aggregates: An effect of mutual interactions. Int. J. Biol. Macromol..

[21] Trombik P., Cieślik-Boczula K. (2020). Influence of phenothiazine molecules on the interactions between positively charged poly-L-lysine and negatively charged DPPC/DPPG membranes. Spectrochim. Acta A Mol. Biomol. Spectrosc..

[22] Cieślik-Boczula K. (2019). Effect of phenothiazine compounds on the secondary structure and fibrillogenesis of poly-L-lysine. Spectrochim. Acta A Mol. Biomol. Spectrosc..

[23] Waeytens J., De Meutter J., Goormaghtigh E., Dazzi A., Raussens V. (2023). Determination of Secondary Structure of Proteins by Nanoinfrared Spectroscopy. Anal. Chem..

[24] D’Arco A., Di Fabrizio M., Mancini T., Mosetti R., Macis S., Tranfo G., Della Ventura G., Marcelli A., Petrarca M., Lupi S. (2023). Secondary Structures of MERS-CoV, SARS-CoV, and SARS-CoV-2 Spike Proteins Revealed by Infrared Vibrational Spectroscopy. Int. J. Mol. Sci..

[25] Novak U., Žerovnik E., Taler-Verčič A., Tušek-Žnidarič M., Zupančič B., Grdadolnik J. (2023). Amyloid Formation of Stefin B Protein Studied by Infrared Spectroscopy. Front. Biosci..

[26] Cieślik-Boczula K. (2017). The PPII-to-α-helix transition of poly-L-lysine in methanol/water solvent mixtures accompanied by fibrillar self-aggregation: An influence of fluphenazine molecules. Biophys. Chem..

[27] Cieślik-Boczula K. (2017). Alpha-helix to beta-sheet transition in long-chain poly-L-lysine: Formation of alpha-helical fibrils by poly-L-lysine. Biochimie.

[28] Giugliarelli A., Sassi P., Paolantoni M., Morresi A., Dukor R., Nafie L. (2013). Vibrational Circular Dichroism Spectra of Lysozyme Solutions: Solvent Effects on Thermal Denaturation Processes. J. Phys. Chem. B.

[29] Pazderková M., Pazderka T., Shanmugasundaram M., Dukor R.K., Lednev I.K., Nafie L.A. (2017). Origin of enhanced VCD in amyloid fibril spectra: Effect of deuteriation and pH. Chirality.

[30] Kurouski D., Lu X., Popova L., Wan W., Shanmugasundaram M., Stubbs G., Dukor R.K., Lednev I.K., Nafie L.A. (2014). Is Supramolecular Filament Chirality the Underlying Cause of Major Morphology Differences in Amyloid Fibrils?. J. Am. Chem. Soc..

[31] Majka Z., Kwiecień K., Kaczor A. (2024). Vibrational Optical Activity of Amyloid Fibrils. ChemPlusChem.

[32] Pescitelli G., Di Bari L. (2024). The Phenomenon of Vibrational Circular Dichroism Enhancement: A Systematic Survey of Literature Data. J. Phys. Chem. B.

[33] Unnikrishnan A.C., Balamurugan K., Shanmugam G. (2023). Structural Insights into the Amyloid Fibril Polymorphism Using an Isotope-Edited Vibrational Circular Dichroism Study at the Amino Acid Residue Level. J. Phys. Chem. B.

[34] Liu D., Chen N., Zhang T., Zhou X., Liu S. (2024). The pH effects on thermal amyloid fibrillation kinetics of hen egg-white lysozyme using new normalization factor for Raman spectroscopy. J. Raman Spectrosc..

[35] Ahlgren K., Havemeister F., Andersson J., Esbjörner E.K., Swenson J. (2024). The inhibition of fibril formation of lysozyme by sucrose and trehalose. RSC Adv..

[36] Magsumov T., Fatkhutdinova A., Mukhametzyanov T., Sedov I. (2019). The Effect of Dimethyl Sulfoxide on the Lysozyme Unfolding Kinetics, Thermodynamics, and Mechanism. Biomolecules.

[37] Sirotkin V.A. (2025). Lysozyme in Mixtures of Hydrogen Bond Acceptor Solvents with Water: Enthalpies and Entropies of Preferential Solvation. J. Phys. Chem. B.

[38] Mukhametzyanov T.A., Fatkhutdinova A.A., Sedov I.A., Yakimova L.S., Klimovitskii A.E. (2021). Calorimetric observation of lysozyme degradation at elevated temperature in water and DMSO-water mixtures. Thermochim. Acta.

[39] Kamiyama T., Liu H.L., Kimura T. (2009). Preferential solvation of lysozyme by dimethyl sulfoxide in binary solutions of water and dimethyl sulfoxide. J. Therm. Anal. Calorim..

[40] Giugliarelli A., Paolantoni M., Morresi A., Sassi P. (2012). Denaturation and preservation of globular proteins: The role of DMSO. J. Phys. Chem. B.

[41] Torreggiani A., Di Foggia M., Manco I., De Maio A., Markarian S.A., Bonora S. (2008). Effect of sulfoxides on the thermal denaturation of hen lysozyme: A calorimetric and Raman study. J. Mol. Struct..

[42] Sassi P., Giugliarelli A., Paolantoni M., Morresi A., Onori G. (2011). Unfolding and aggregation of lysozyme: A thermodynamic and kinetic study by FTIR spectroscopy. Biophys. Chem..

[43] Gremlich H.U., Yan B. (2001). Infrared and Raman Spectroscopy of Biological Materials.

[44] Kong J., Yu S. (2007). Fourier transform infrared spectroscopic analysis of protein secondary structures. Acta Biochim. Biophys. Sin..

[45] Shariatizi S., Meratan A.A., Ghasemi A., Nemat-Gorgani M. (2015). Inhibition of amyloid fibrillation and cytotoxicity of lysozyme fibrillation products by polyphenols. Int. J. Biol. Macromol..

[46] Zaidi F.K., Bhat R. (2018). Resveratrol Interferes with an Early Step in the Fibrillization Pathway of Human Lysozyme and Modulates It Toward Less-Toxic, Off-Pathway Aggregates. ChemBioChem.

